# A SNP profiling panel for sample tracking in whole-exome sequencing studies

**DOI:** 10.1186/gm492

**Published:** 2013-09-27

**Authors:** Reuben J Pengelly, Jane Gibson, Gaia Andreoletti, Andrew Collins, Christopher J Mattocks, Sarah Ennis

**Affiliations:** 1 Human Genetics and Genomic Medicine, Faculty of Medicine, University of Southampton, Duthie Building (MP 808), Southampton General Hospital, Tremona Road, Southampton SO16 6YD, UK; 2 National Genetics Reference Laboratory (Wessex), Salisbury District Hospital, Salisbury SP2 8BJ, UK

## Abstract

Whole-exome sequencing provides a cost-effective means to sequence protein coding regions within the genome, which are significantly enriched for etiological variants. We describe a panel of single nucleotide polymorphisms (SNPs) to facilitate the validation of data provenance in whole-exome sequencing studies. This is particularly significant where multiple processing steps necessitate transfer of sample custody between clinical, laboratory and bioinformatics facilities. SNPs captured by all commonly used exome enrichment kits were identified, and filtered for possible confounding properties. The optimised panel provides a simple, yet powerful, method for the assignment of intrinsic, highly discriminatory identifiers to genetic samples.

## Background

Whole-exome sequencing (WES) is presently one of the most efficient means of identifying aetiological genetic mutations [1], minimising some of the challenges associated with whole-genome sequencing, such as high cost and data processing burden, analysis and interpretation. In WES, protein-coding regions of the genome are targeted and enriched via specific hybridisation of genomic fragments with complementary oligonucleotides, or 'baits’. These targeted regions are then sequenced using high throughput next-generation sequencing (NGS) technologies [2].

The high start-up investment required for in-house WES is currently prohibitive to many groups so sample preparation and/or sequencing is commonly outsourced. This transference of sample custody, combined with the complex sample preparation workflow, makes sample mix-ups possible, and difficult to detect. In both clinical and research contexts, ensuring provenance of data is essential to allow the accurate assignment of clinical details to sequence data. It is possible that samples may be misidentified at any stage of the analytical process, both *in vitro* and *in silico*. Therefore, sample tracking must be contiguous throughout both data generation and analysis.

Consequent to sample mix-ups in a research setting, erroneous data and sample matching may result in a loss of power for identification of causal variants [3]. In a clinical setting, this may lead to delayed or inaccurate reporting of results to patients. Whilst good practice in the handling of samples and increased laboratory automation minimises potential for error, additional checkpoints are still required to support quality control [4]. A method for *post hoc* confirmation of sample identity is therefore highly desirable.

Genetic sample identification methods have an advantage over alternative sample management systems in that the genetic 'label’ is intrinsic to the biological sample itself, removing the possibility of manual labelling errors. Single nucleotide polymorphisms (SNPs) are increasingly utilised for DNA-based identification of human samples, with several benefits compared to standard forensic methods [5-7]. Existing SNP panels for human forensic identification and commercial SNP panels for sample identification, such as the iPLEX Sample ID Plus panel (Sequenom, San Diego, CA, USA), utilise pan-genome SNPs, the majority of which are non-exonic, and are therefore not useful for WES studies, as the majority of markers will not lie within the enriched regions of the genome. In addition to existing SNP panels, short tandem repeat markers can be used for genetic sample tracking. However, again, markers applied are frequently outside exomic regions and, if captured, will be prone to erroneous NGS genotyping using standard pipelines due to the repetitive nature of the markers [8].

Several methods for genetic tracking of human biological samples have been previously described, some of which are application specific - for example, for transcriptome microarray studies [3,9,10]. Although software for the validation of NGS (including WES) sample identity, such as verifyBamID, exist [11], for the detection of sample misidentifications external array-based genotypes of the samples are required, without which only contamination of the samples can be assessed.

Here we describe an optimised panel of SNPs for which WES data are typically informative, the genotypic profile of which can be utilised to extract intrinsic identifiers from human genomic DNA. These SNP profiles have high discriminatory power, even in large datasets. The profile derived from this panel can be compared to an independently genotyped profile for the same individual, allowing accurate validation of data and sample pairings, at a modest cost per sample.

## Methods

### Candidate identification and panel selection

Regions of overlap between three current commonly used whole-exome enrichment kits, (namely Agilent SureSelect Human All Exon V4, Illumina TruSeq Exome Enrichment and Nimblegen SeqCap EZ Human Exome Library V3.0 kits), and common SNPs (dbSNP 137, [12]) were established using BEDTools [13]. SNPs were further filtered for inclusion based upon their presence in genes targeted by the Illumina TruSight Exome kit, which targets only genes of clinical interest.

Primary candidate selection criteria required SNPs to: 1) represent bi-allelic substitutions, excluding substitutions of complementary bases, that is, A↔T and G↔C transversions; 2) be technically amenable to both accurate WES and orthogonal genotyping, that is, not present in large-scale genomic repeats [14], or homopolymeric tracts of ≥5 bp, GC content for the flanking 250 bp was restricted to a range of between 40% and 55% and no other variant within 50 bp with an alternative-allele frequency (AF) ≥0.01 was permitted; 3) conform to desirable phase 3 HapMap AFs across several populations, explicitly AFs of between 0.2 and 0.8 in: CEPH (Utah residents with ancestry from northern and western Europe; CEU), Japanese in Tokyo, Japan (JPT), Han Chinese in Beijing, China (CHB) and Yoruba in Ibidan, Nigeria (YRI) [15] and; 4) not alter the primary sequence of the encoded protein or have an associated Online Mendelian Inheritance in Man (OMIM) record [16].

Following primary candidate identification steps, SNPs were further optimised by the following requirements: 1) be located at least 10 bp from exon boundaries; 2) not be situated in regions with a high sequence similarity to non-target regions, that is, no non-target BLAT score >100 [17], as this could result in non-specific genotyping; and 4) be outside of linkage disequilibrium with all other selected SNPs.

Finally, SNPs were prioritised for inclusion in the panel by proximity of the AFs to 0.5, across HapMap populations, in order to maximise discriminatory power.

### SNP coverage in whole-exome sequencing data

A set of 91 in-house exome samples was evaluated for depth of sequence coverage for the candidate SNPs. Exome capture was performed using Agilent SureSelect Human All Exon V3 (n = 22) and V4 (n = 55), Illumina TruSeq Exome Enrichment (n = 9) and Nimblegen SeqCap EZ Human Exome Library V3.0 (n = 5). Exome enrichment, sequencing and *in silico* analysis of samples was performed as previously described [18,19].

### Optimised panel validation

The power of sample resolution for the panel was validated using data from phase 1 of the 1000 Genomes Project (n = 1,092) [20] and the UK10K project (n = 2,688; 2,432 of which are whole-genome data) [21]. Genotypes were extracted from data using custom scripts and Tabix [22]. Quantification of mismatches between samples was performed using MEGA5 [23].

Simulated datasets were generated by taking individual population AFs for each SNP as input and generating random SNP profiles in accordance with Hardy-Weinberg equilibrium based upon this; the randomisation of each SNP was independent of all other SNPs. We then quantified the rate of non-unique profiles per simulated dataset. We performed 20,000 independent replicates of dataset generation in all cases.

### Panel application

We applied the panel to a batch of 48 samples exome sequenced by an external service provider, for which orthogonal genotypes were obtained concurrently through an independent genotyping provider using KASP genotyping (LGC Genomics, Hoddeston, UK). Following plating of DNA samples for dispatch, a replicate plate was made directly from the primary plate, to be dispatched for the orthogonal genotyping. Genotypes derived from exome data and orthogonal genotyping assays were compared using PLINK [24] and custom scripts.

### Ethics

This study was approved by the Southampton and South West Hampshire Research Ethics Committee (09/H0504/125). Informed consent was obtained for all participants.

## Results

**Figure 1:**
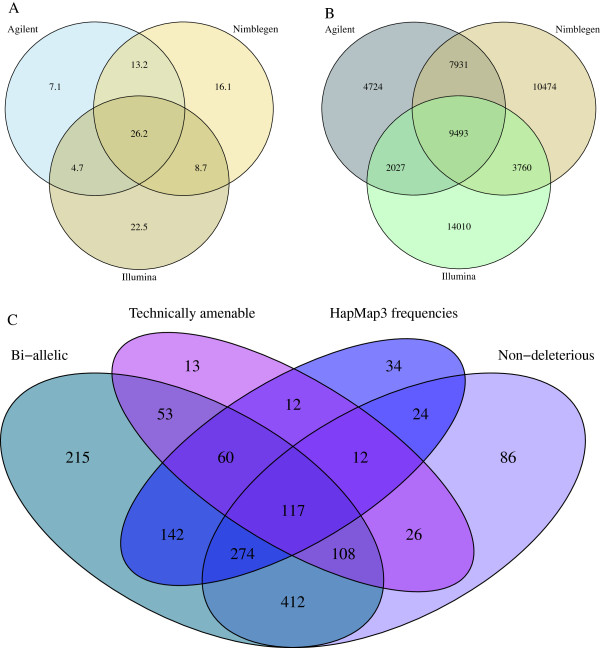
**Venn diagrams showing commonality of targeting between capture kits (A,B) and properties of encompassed SNPs (C).** Overlap between exome capture kits is presented in Mbp **(A)** and number of SNPs with an AF ≥0.3 **(B)**. Agilent - SureSelect Human All Exon V4; Illumina - TruSeq Exome Enrichment; Nimblegen - SeqCap EZ Human Exome Library V3.0. For a subset of SNPs present in both the intersection of the three kits shown, and the Illumina TruSight Exome kit, a breakdown of fulfilment of the four classes of candidate filtering criteria is shown **(C)** (see the main text for details of filtering criteria); 117 SNPs exhibited all desired characteristic; 74 SNPs exhibited none of the desired characteristics.

In total, 26.2 Mbp of genome sequence was found to overlap all three commonly applied whole-exome capture kits, containing 9,493 common SNPs (Figure 1A,B). Of these, 1,662 SNPs are additionally covered by the Illumina TruSight Exome kit. Within this subset, following the filtering for all primary candidate criteria, 117 candidate SNPs were identified (Figure 1C; Additional file 1), from which the optimised panel of 24 SNPs was selected (Table 1). Within the set of 91 in-house exome samples, all 24 SNPs were sequenced at sufficient read-depth for accurate genotype calling, across all capture kits.

**Table 1:** Optimised panel of identifying SNPs

**Chromosome**	**Position**^a^	**dbSNP rsID**	**Gene**	**Alleles**	**HapMap 3 AF**			
					**CEU**	**CHB**	**JPT**	**YRI**
1	179520506	rs1410592	*NPHS2*	A/C	0.59	0.62	0.54	0.53
1	67861520	rs2229546	*IL12RB2*	A/G	0.64	0.36	0.44	0.58
2	169789016	rs497692	*ABCB11*	A/G^b^	0.55	0.65	0.51	0.22
2	227896976	rs10203363	*COL4A4*	C/T	0.46	0.44	0.36	0.57
3	4403767	rs2819561	*SUMF1*	A/G^b^	0.56	0.73	0.73	0.72
4	5749904	rs4688963	*EVC*	A/G^b^	0.33	0.65	0.67	0.52
5	82834630	rs309557	*VCAN*	A/G^b^	0.49	0.34	0.52	0.50
6	146755140	rs2942	*GRM1*	C/T	0.54	0.49	0.55	0.47
7	48450157	rs17548783	*ABCA13*	C/T	0.46	0.72	0.53	0.48
8	94935937	rs4735258	*PDP1*	C/T	0.40	0.64	0.66	0.46
9	100190780	rs1381532	*TDRD7*	A/G^b^	0.48	0.59	0.50	0.58
10	100219314	rs10883099	*HPSE2*	A/G	0.52	0.52	0.53	0.62
11	16133413	rs4617548	*SOX6*	C/T	0.52	0.65	0.61	0.51
12	993930	rs7300444	*WNK1*	A/G	0.46	0.55	0.48	0.28
13	39433606	rs9532292	*FREM2*	A/G	0.29	0.41	0.44	0.54
14	50769717	rs2297995	*L2HGDH*	A/G	0.55	0.65	0.67	0.59
15	34528948	rs4577050	*SLC12A6*	C/T	0.68	0.75	0.63	0.32
16	70303580	rs2070203	*AARS*	A/G^b^	0.53	0.28	0.51	0.49
17	71197748	rs1037256	*COG1*	C/T	0.50	0.67	0.65	0.56
18	21413869	rs9962023	*LAMA3*	A/G	0.67	0.81^c^	0.75	0.51
19	10267077	rs2228611	*DNMT1*	C/T^b^	0.47	0.73	0.56	0.48
20	6100088	rs10373	*FERMT1*	G/T^b^	0.54	0.31	0.35	0.58
21	44323590	rs4148973	*NDUFV3*	C/T	0.65	0.33	0.38	0.73
22	21141300	rs4675	*SERPIND1*	A/C	0.46	0.62	0.51	0.57

^a^Position as defined in genome reference assembly GRCh37 (hg19).

^b^SNP is defined on the negative strand.

^c^AF marginally outside target range for candidate selection. Selected due to paucity of candidates on chromosome 18.

The 24 biallelic SNPs afford 48 points of allelic comparison. Testing the optimised panel in the 1000 Genomes Project data (n = 1,092) [20], an average of 18.0 (standard deviation = 3.3) allelic differences between all pairwise combinations was observed, with a range of 3 to 34. As such, there will be, on average, 18 differential alleles between any two samples, enabling discrimination.

On addition of the UK10K data (n = 2,688) to the 1000 Genomes Project data (n_combined_ = 3,780), there remained an average of 17.8 allele mismatches across the profiles. Eighteen UK10K sample pairs produced duplicate profiles. On investigation of these pairs, they were found to share >98% genotypic concordance across an extended panel of 1,662 SNPs in all cases, compared to an average of 42%, with a range of 27 to 77%, for all sample pairs with unique SNP profiles (Additional file 2). As such, these pairs represent extreme outliers, and are derived from genetically identical biological samples, either from the same individual or monozygotic twins, and were therefore excluded from the mismatch average.

### Simulated data

The discriminatory power of the panel was evaluated by dataset simulation. We simulated datasets of 10,000 individuals, that conformed to AF distributions for investigated HapMap populations (CEU, CHB, JPT and YRI), 1000 Genomes Project pilot average [25], as well as for a hypothetical perfect allele distribution (AF = 0.5 for all SNPs) (Table 2). In all simulated populations, <2.5% of simulated datasets of 10,000 contained any repeat SNP profiles (henceforth termed 'collisions’). This translates approximately into less than 1 in every 40 independent datasets of 10,000 individuals containing a single matching pair of profiles.

**Table 2:** Profile collisions per simulated dataset of 10,000 individuals with various population AFs

**AF source**	**Average collisions per dataset (±SD)**
**1000 Genomes average**	0.0039 (0.062)
**HapMap phase 3:**	
CEU	0.0064 (0.079)
CHB	0.0239 (0.154)
JPT	0.0082 (0.090)
YRI	0.0076 (0.086)
**Theoretical perfect**^**a**^	0.0031 (0.056)

^a^All 24 SNPs assigned an AF of 0.5, which will give the most even trifurcation per SNP, and thus discriminatory power. SD, standard deviation.

**Figure 2:**
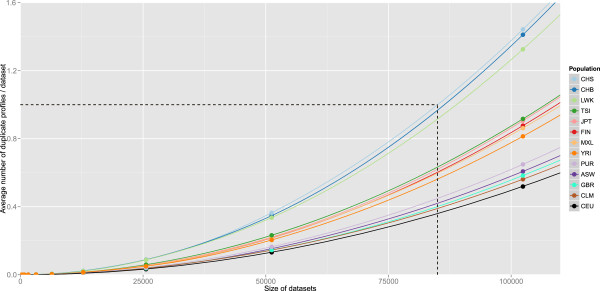
**Relationship between size of simulated datasets and the occurrence of non-unique profiles.** Thirteen 1000 Genomes Project populations were simulated [20]. Datasets were simulated as described in Methods. With increasing dataset size, the probability of repeat profiles increases. Only populations with a sample size of >50 individuals in the dataset were simulated. Additional populations are Americans of African ancestry in Southwest USA (ASW), Columbians from Medellin, Colombia (CLM), Finnish in Finland (FIN), British in England and Scotland (GBR), Luhya in Webuye, Kenya (LWK), Mexican ancestry from Los Angeles, USA (MXL), Puerto Ricans from Puerto Rico (PUR) and Toscany in Italia (TSI).

The effect of dataset size on the frequency of collisions was investigated for populations present in 1000 Genomes Project phase 1 data [20]. An exponential increase in the frequency of collisions was observed with increasing dataset size, though the panel continued to have high power for the discrimination of samples. For instance, were we to have 85,000 Southern Han Chinese (CHS) samples, (the worst performing 1000 Genomes population evaluated, due to the AF distribution for SNPs within this panel), we would expect the dataset to contain, on average, a single duplicate SNP profile (Figure 2). In addition, total SNP absence - for example, through technical failure of orthogonal genotyping - was modelled. For each SNP that entirely failed to provide data, a less than three-fold drop in discriminatory power was observed in all cases (data not shown). This suggests that our approach is robust against technical failure.

**Figure 3:**
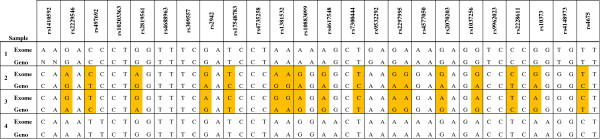
**Exome derived and orthogonal genotypes (Geno) for four samples, showing a sample-switch between samples 2 and 3.** Informative markers for the resolution of this switch are highlighted in yellow.

Application of the SNP panel to our batch of 48 samples revealed a discrepancy between exome and orthogonal genotypes for two samples dispatched in adjacent wells, suggesting a reciprocal transposition (Figure 3). The occurrence of this error in the exome data was also supported by interrogation of X-chromosome heterozygosity to confirm sample gender. In addition to the identification of the switch, the panel allowed for expeditious resolution of the error, permitting the continued use of the data in downstream analyses.

## Discussion

Validation of sample identity is essential in order to ensure data integrity and validity of conclusions drawn from data. We have described a powerful tool for the identification and validation of data provenance throughout the workflow of WES data collection and analysis. The power of discrimination, that is, the precision with which samples can be uniquely identifiable, is sufficient and robust for most projects on the current scale of up to 10,000 samples, with inbuilt redundancy of SNPs to protect against technical failures. In WES, the exome enrichment process provides the limiting step for the availability of data on SNPs for use in sample identification. As such, this panel will also be of utility for whole-genome sequencing data, where there is no such limitation on SNP coverage. This will be beneficial where there are mixed datasets of both whole-genome sequence and WES data.

NGS is now developing as the diagnostic methodology of choice across a range of applications, including mutation scanning in targeted gene panels and WES for congenital disorders, as well as high depth analysis for tumour profiling. Whilst the service model for delivery of these tests is not fully resolved at this stage, there will certainly be economic arguments for centralising certain tests. This will have the effect of increasing the throughput requirements as well as physically moving samples between labs. Both of these factors will increase the opportunity for sample misidentification.

Even for testing within a single lab, the use of inherent sample and data identification methods, as described in this study, seems a robust approach to fulfil the regulatory requirement for providing a full audit trail and ensuring data provenance [26,27]. The SNP panel presented here is immediately usable across all commonly used exome capture kits, and would be equally applicable to any gene panel by incorporating, or 'spiking’, the SNP regions into the custom capture kit at the design stage. Where it can be shown that there are no expected repeat profiles (that is, no paired samples from the same individual are being analysed), it may even be beneficial from a process perspective to use the SNP profile as the primary method for sample tracking.

The discriminatory power of the panel may be reduced for various reasons, such as geographically localised variation in AFs, and degradation of DNA samples, resulting in incomplete data. We have shown our panel to have a high discriminatory power across a diverse range of populations. Additionally, the discriminatory power will be marginally reduced where many relatives are sequenced. In the case of highly consanguineous families, sample tracking methods such as barcoding will afford optimal certainty in these particular cases. Should concerns over insufficient discriminatory power arise, additional SNPs may be added to the panel from the existing list of candidates (Additional file 1), also allowing the tailoring of an enhanced panel to the population(s) of interest, should this be desired. Nevertheless, we have demonstrated our panel to be sufficiently robust to withstand power reductions without loss of utility for most purposes.

We have also presented a recent case in which use of this panel has allowed us to identify, confirm, and resolve a sample switch, highlighting the importance of using such a tool. Monetary cost will vary with the technology used for orthogonal genotyping and sample throughput. We have intentionally designed the panel to be platform non-specific, allowing for the establishment of in-house assays using preferred genotyping methodology or outsourced where required. Our own chosen methodology costs approximately £5 GBP per sample, representing a small fraction of the cost of exome data generation.

## Conclusions

The size of held NGS datasets continues to increase, with the UK Government recently committing to the sequencing of 100,000 samples as part of healthcare provisions [28]. As such, the demand for the development of effective tools for bioinformatic analysis, data compression, mutation effect prediction and quality control is high. We have described a panel of SNPs for the discrimination of human biological samples on the basis of data intrinsic to WES data derived from samples processed using common capture kits. We recommend the routine use of this panel to maintain data integrity and protect sample provenance.

## Abbreviations

AF: alternative-allele frequency; bp: base pair; CEU: CEPH (Utah residents with ancestry from northern and western Europe); CHB: Han Chinese in Beijing, China; CHS: Southern Han Chinese; JPT: Japanese in Tokyo, Japan; Mbp: megabase pair; NGS: next-generation sequencing; SNP: single nucleotide polymorphism; WES: whole-exome sequencing; YRI: Yoruba in Ibidan, Nigeria.

## Competing interests

The authors declare that they have no competing interests.

## Authors’ contributions

RJP performed analysis and interpretation of data, and drafted the manuscript, JG, GA and AC contributed to analysis, CJM contributed to data interpretation and manuscript preparation and SE conceived and supervised the project, and contributed to manuscript preparation. All authors read and approved the final manuscript.

## Supplementary Material

Additional file 1 List of all candidate SNPs with evaluated properties. Click here for file

Additional file 2 **Distribution of pairwise genotype concordance between samples.** Pairs resulting in duplicate SNP profiles (n = 18) and pairs between samples with unique SNP profiles (n = 7,142,293) within the combined dataset of 3,780 samples are shown. Concordance across the 1,662 SNPs detailed in Figure 1C was evaluated. All pairs resulting in duplicate profiles have >98% concordance, well separated from the distribution of samples with unique profiles. Note the logarithmic scale. Click here for file
