# Erratum to: a SNP profiling panel for sample tracking in whole-exome sequencing studies

**DOI:** 10.1186/s13073-015-0163-1

**Published:** 2015-05-07

**Authors:** Reuben J Pengelly, Jane Gibson, Gaia Andreoletti, Andrew Collins, Christopher J Mattocks, Sarah Ennis

**Affiliations:** Human Genetics and Genomic Medicine, Faculty of Medicine, University of Southampton, Duthie Building (MP 808), Southampton General Hospital, Tremona Road, Southampton, SO16 6YD UK; National Genetics Reference Laboratory (Wessex), Salisbury District Hospital, Salisbury, SP2 8BJ UK

## Abstract

This is an Erratum to Genome Medicine 2013, 5:89, highlighting an error in Table 1 of the original article. Please see related article: http://genomemedicine.com/content/5/9/89.

## Erratum

It has come to our attention that there is an error in Table 1 of our article [1]. Within the ‘Alleles’ column, incorrect reference/alternate nucleotides have been given. This error is limited to this column, all other data and conclusions presented are correct. The corrected Table 1 is shown below.

**Table 1:** **Optimised panel of identifying SNPs**

**Chromosome**	**Position** ^**a**^	**dbSNP rsID**	**Gene**	**Alleles**	**HapMap 3 AF**			
					**CEU**	**CHB**	**JPT**	**YRI**
1	179520506	rs1410592	*NPHS2*	A/G	0.59	0.62	0.54	0.53
1	67861520	rs2229546	*IL12RB2*	A/C	0.64	0.36	0.44	0.58
2	169789016	rs497692	*ABCB11*	A/G^b^	0.55	0.65	0.51	0.22
2	227896976	rs10203363	*COL4A4*	C/T	0.46	0.44	0.36	0.57
3	4403767	rs2819561	*SUMF1*	C/T^b^	0.56	0.73	0.73	0.72
4	5749904	rs4688963	*EVC*	A/G^b^	0.33	0.65	0.67	0.52
5	82834630	rs309557	*VCAN*	A/G^b^	0.49	0.34	0.52	0.50
6	146755140	rs2942	*GRM1*	A/G	0.54	0.49	0.55	0.47
7	48450157	rs17548783	*ABCA13*	C/T	0.46	0.72	0.53	0.48
8	94935937	rs4735258	*PDP1*	C/T	0.40	0.64	0.66	0.46
9	100190780	rs1381532	*TDRD7*	C/T^b^	0.48	0.59	0.50	0.58
10	100219314	rs10883099	*HPSE2*	A/G	0.52	0.52	0.53	0.62
11	16133413	rs4617548	*SOX6*	A/G	0.52	0.65	0.61	0.51
12	993930	rs7300444	*WNK1*	C/T	0.46	0.55	0.48	0.28
13	39433606	rs9532292	*FREM2*	A/G	0.29	0.41	0.44	0.54
14	50769717	rs2297995	*L2HGDH*	A/G	0.55	0.65	0.67	0.59
15	34528948	rs4577050	*SLC12A6*	A/G	0.68	0.75	0.63	0.32
16	70303580	rs2070203	*AARS*	C/T^b^	0.53	0.28	0.51	0.49
17	71197748	rs1037256	*COG1*	A/G	0.50	0.67	0.65	0.56
18	21413869	rs9962023	*LAMA3*	C/T	0.67	0.81^c^	0.75	0.51
19	10267077	rs2228611	*DNMT1*	A/G^b^	0.47	0.73	0.56	0.48
20	6100088	rs10373	*FERMT1*	C/T^b^	0.54	0.31	0.35	0.58
21	44323590	rs4148973	*NDUFV3*	G/T	0.65	0.33	0.38	0.73
22	21141300	rs4675	*SERPIND1*	C/T	0.46	0.62	0.51	0.57

^a^Position as defined in genome reference assembly GRCh37 (hg19).

^b^SNP alleles are defined on the negative strand to be consistent with dbSNP.

^c^AF marginally outside target range for candidate selection. Selected due to paucity of candidates on chromosome 18.
