# Supplementary figures and images for: A SNP profiling panel for sample tracking in whole-exome sequencing studies
Source: Genome Med. 2013 Sep 27;5(9):89. doi: 10.1186/gm492 (PMC3978886; doi:10.1186/gm492)

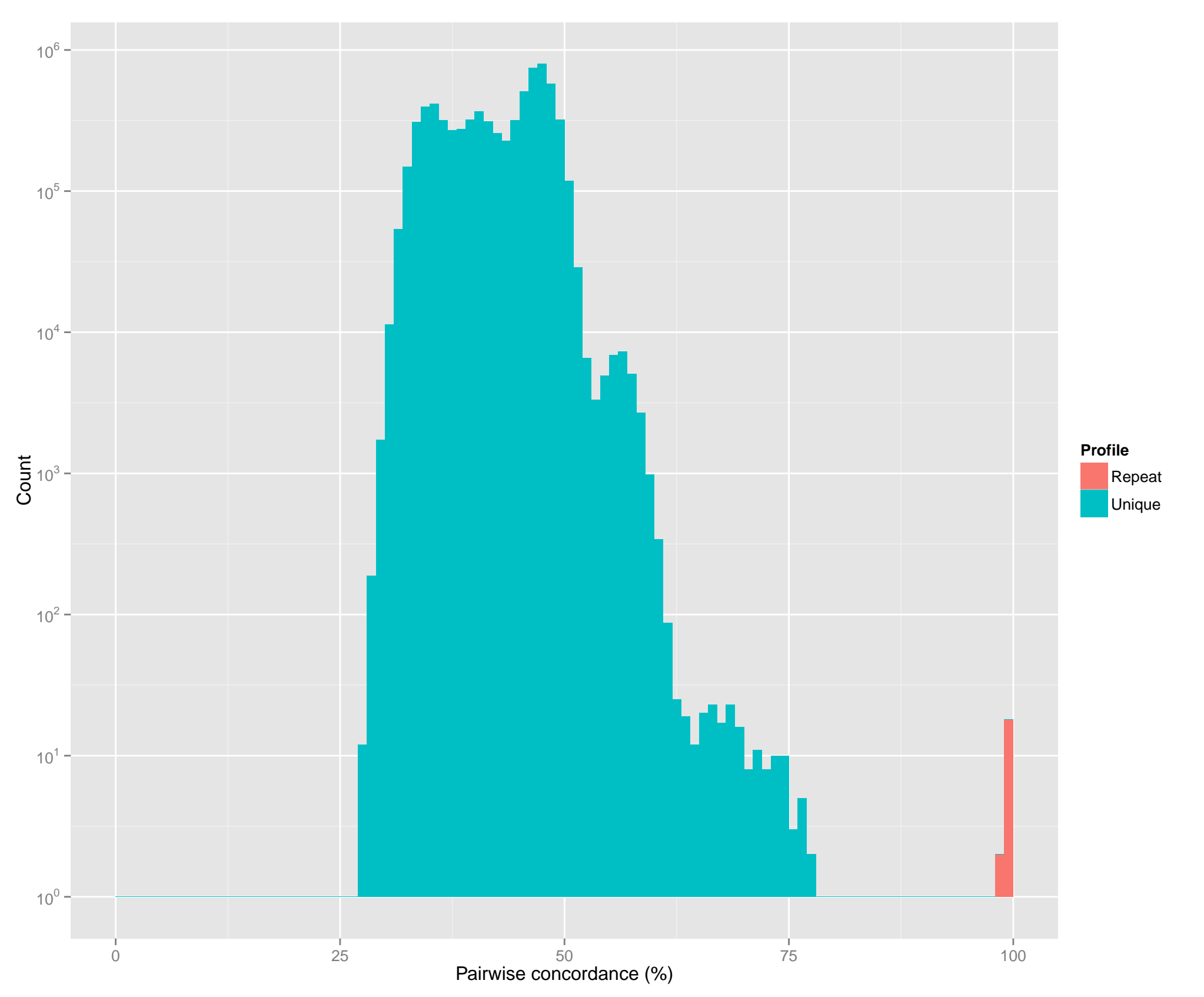

Supplement: Additional file 2 — Distribution of pairwise genotype concordance between samples. Pairs resulting in duplicate SNP profiles (n = 18) and pairs between samples with unique SNP profiles (n = 7,142,293) within the combined dataset of 3,780 samples are shown. Concordance across the 1,662 SNPs detailed in Figure 1C was evaluated. All pairs resulting in duplicate profiles have >98% concordance, well separated from the distribution of samples with unique profiles. Note the logarithmic scale. [file gm492-S2.pdf]
